# Physiotherapy Management of Plantar Fasciitis: A National Cross-Sectional Survey in Saudi Arabia

**DOI:** 10.3390/jcm14134584

**Published:** 2025-06-27

**Authors:** Abdulmajeed Muhaysin Alnefaie, Hosam Alzahrani, Mansour Abdullah Alshehri

**Affiliations:** 1 Department of Medical Rehabilitation and Physiotherapy, Eradah Complex and Mental Health—Taif Health Cluster, Taif 26513, Saudi Arabia; aalnufaiei@moh.gov.sa; 2 Department of Physical Therapy, College of Applied Medical Sciences, Taif University, Taif 21944, Saudi Arabia; halzahrani@tu.edu.sa; 3 Department of Medical Rehabilitation Sciences, Faculty of Applied Medical Sciences, Umm Al-Qura University, Mecca 24382, Saudi Arabia

**Keywords:** plantar fasciitis, heel pain, physiotherapy, physiotherapists, rehabilitation, clinical practice guideline, Saudi Arabia

## Abstract

**Background/Objectives**: Plantar fasciitis is the most common cause of heel pain, affecting 4–7% of the general population. Physiotherapy is a key component of conservative management. However, there is limited evidence on how physiotherapists in Saudi Arabia manage this condition. This study aimed to investigate current physiotherapy practices for plantar fasciitis in Saudi Arabia and assess their alignment with international clinical guidelines. **Methods**: A cross-sectional survey was conducted among licensed physiotherapists practicing in Saudi Arabia who had treated patients with plantar fasciitis. An online questionnaire, adapted from a validated UK-based survey, gathered data on participant demographics, service characteristics, diagnostic criteria, treatment goals, outcome measures, and intervention strategies. Descriptive statistics were used for analysis. **Results**: A total of 399 physiotherapists participated. Diagnosis was mainly based on clinical signs such as pain during plantar fascia stretch (72.9%), early morning pain (70.4%), and medial heel tenderness (69.4%). Common goals of intervention included pain reduction (93.4%), functional improvement (69.9%), and patient education (57.3%). Pain scales (74.9%) and functional tests (49.1%) were the most frequently used outcome measures. Interventions such as exercise therapy (92.0%), stretching (89.4%), and strengthening (84.7%) were widely used. More advanced modalities like shockwave therapy and dry needling were less frequently reported. **Conclusions**: Physiotherapy practices largely align with international guidelines. However, variation in outcome assessments and underuse of advanced modalities indicate the need for national clinical guidelines and targeted training programs. These steps may promote more consistent, evidence-based care and improve patient outcomes in Saudi Arabia.

## 1. Introduction

Plantar fasciitis is the most common cause of inferior heel pain, accounting for nearly 80% of heel pain cases and affecting approximately 4% to 7% of the general population [1]. It typically affects adults between 40 and 60 years of age and is more frequently observed in women [2,3]. Clinically, it presents as sharp, localized pain at the medial heel, especially during the first steps in the morning or after periods of rest. This presentation is often accompanied by biomechanical impairments such as limited ankle dorsiflexion, tightness in the posterior chain muscles, and altered gait mechanics [2,4].

The condition is degenerative in nature and results from repetitive micro-trauma to the plantar fascia. Its aetiology is multifactorial, with established risk factors including obesity, prolonged standing, inappropriate footwear, reduced flexibility, and participation in high-impact physical activities such as running [4,5,6,7]. These risk factors are especially relevant in Saudi Arabia, where national data indicate that over 35% of adults are classified as obese and more than 60% do not meet recommended levels of physical activity [8,9]. Additionally, large segments of the workforce, including healthcare professionals, educators, and retail employees, spend extended hours standing, which may further increase the risk of plantar fascia-related disorders [10].

Plantar fasciitis is mainly diagnosed through a patient’s history and physical examination. Patients commonly report pain with the first steps in the morning, while clinical signs include localized tenderness at the medial calcaneal tubercle and limited ankle dorsiflexion [2,11]. The examination also includes assessing gait, muscle strength, and heel cord tightness [11,12]. It is important to rule out other causes of heel pain, such as fat pad atrophy, calcaneal stress fracture, or tarsal tunnel syndrome [2,11]. Imaging is not always required but can support the diagnosis when needed [2,11,13]. Ultrasound is commonly used because it is safe, affordable, and effective in measuring plantar fascia thickness and guiding treatment [13]. MRI may be used in more complex or unclear cases to rule out other conditions [2,11].

Conservative management is the primary treatment approach, with physiotherapy playing a central role. A range of evidence-based interventions is recommended, including stretching exercises for the plantar fascia and calf muscles, strengthening of foot and hip musculature, taping techniques, orthotic support, manual therapy, dry needling, and extracorporeal shockwave therapy [11,12,14,15]. Clinical practice guidelines developed by the Orthopaedic Section of the American Physical Therapy Association support a multimodal approach that combines therapeutic exercise with manual therapy to improve clinical outcomes [16]. When applied effectively, these interventions lead to symptom resolution in the majority of patients [4,17].

Despite the availability of high-quality evidence and guidelines, clinical practice often varies across different healthcare systems. For example, a national survey in the United Kingdom reported that physiotherapists frequently emphasized education and stretching but made limited use of interventions such as manual therapy and shockwave therapy [18]. That UK-based study reflected practice patterns within a well-established healthcare system supported by the National Health Service, where access to postgraduate training, clinical technologies, and structured professional development is consistently available. In contrast, the physiotherapy profession in Saudi Arabia is evolving within a healthcare system undergoing major reforms [19]. Institutional resources, training opportunities, and access to advanced technologies may differ across regions and clinical settings. To date, no national-level survey has examined how physiotherapists assess and manage plantar fasciitis in the Middle East, highlighting an important gap in the regional literature. Such differences in clinical practice may be influenced by variations in training, resource availability, and healthcare delivery models.

In the context of Saudi Arabia, limited access to continuing education, inconsistencies in postgraduate training, and unequal availability of clinical guidelines and technologies have been identified as systemic challenges facing physiotherapists [20,21]. These factors may contribute to variability in practice and hinder the consistent application of evidence-based care. High rates of musculoskeletal pain among Saudi working populations, such as healthcare workers, office staff, and schoolteachers, further underscore the need to strengthen musculoskeletal care delivery [22,23,24]. This knowledge gap is particularly significant within the broader framework of Saudi Arabia’s Vision 2030 healthcare transformation, which aims to expand allied health services and promote the use of evidence-based clinical practices [19].

Although international guidelines provide clear recommendations, the extent to which physiotherapists in Saudi Arabia follow these recommendations in routine practice remains unclear. Understanding current practice patterns is essential for evaluating alignment with best-practice standards, identifying gaps in care, and informing targeted improvements in clinical training and health policy. Therefore, this study aims to investigate the assessment and management strategies employed by Saudi physiotherapists in treating plantar fasciitis and to evaluate the extent to which their practices reflect current evidence-based recommendations.

## 2. Materials and Methods

### 2.1. Study Design and Ethical Considerations

This study employed a cross-sectional design and was conducted in accordance with the Strengthening the Reporting of Observational Studies in Epidemiology (STROBE) guidelines. Ethical approval was obtained from the Research Ethics Committee at Taif University, Saudi Arabia (Approval No. 44-015). All procedures involving human participants adhered to the principles outlined in the Declaration of Helsinki. Prior to participation, all participants provided informed consent electronically after reviewing a detailed study information sheet embedded in the online questionnaire. Participation was voluntary, and participants could withdraw at any time without consequence. All responses were collected anonymously, with no personally identifying information (e.g., names, IP addresses, or contact details) recorded, thereby ensuring both anonymity and data confidentiality.

### 2.2. Participants and Eligibility Criteria

The study targeted licensed physiotherapists currently practicing in Saudi Arabia who had encountered and/or treated patients with plantar fasciitis in their clinical practice. Inclusion criteria were as follows: (1) being a licensed physiotherapist currently employed in a clinical role within Saudi Arabia, and (2) having personally managed at least one patient with plantar fasciitis. There were no restrictions based on sex, nationality, or healthcare sector. Exclusion criteria included physiotherapists not currently engaged in clinical practice (e.g., full-time academics or administrators), students or interns who were not yet licensed, and individuals without any prior clinical involvement in the assessment or treatment of plantar fasciitis. Initial invitations were distributed through professional physiotherapy networks, institutional mailing lists, and social media platforms such as WhatsApp and X (formerly Twitter). Recipients were encouraged to forward the survey to other eligible colleagues to enhance response rates and ensure broader geographical coverage.

### 2.3. Questionnaire Development and Content

A structured, self-administered questionnaire was developed using the Google Forms platform. Parts 2 to 6 of the questionnaire were taken directly from a previously validated tool developed by Grieve and Palmer [18], originally used in a UK-wide survey of physiotherapists managing plantar fasciitis. As all physiotherapy education programs in Saudi Arabia are delivered in English, and English is the primary language used in clinical documentation and communication among physiotherapists, the original English version was used without translation or cultural adaptation. The only modifications made were to the demographic and practice-related part, which were adjusted to reflect the Saudi healthcare context (e.g., type of institution and years of local experience). The questionnaire comprised six parts:

- Participants’ characteristics (part 1): Collected demographic and professional data, including age, sex, nationality, clinical rank, years of experience, and primary workplace setting.
- Description of physiotherapy services (part 2): Captured information on service accessibility, referral patterns, and average duration of assessment sessions.
- Diagnostic criteria (part 3): Explored the clinical criteria commonly used by participants to diagnose plantar fasciitis.
- Goals of intervention (part 4): Assessed participants’ perspectives on the aims of physiotherapy in managing plantar fasciitis.
- Assessment of outcome measures (part 5): Identified the tools and instruments used to evaluate treatment outcomes.
- Intervention strategies (part 6): Identified the types and frequency of conservative physiotherapy interventions commonly employed in clinical practice.

### 2.4. Sample Size Estimation

A sample size calculation was performed using the Calculator.net sample size calculator. Based on the total number of licensed physiotherapists (n = 12,544) registered with the Saudi Commission for Health Specialties in Saudi Arabia, a minimum sample of 373 participants was required to achieve a 95% confidence level and a 5% margin of error.

### 2.5. Data Analysis

Data were exported to IBM SPSS Statistics for Windows, Version 23.0 (IBM Corp., Armonk, NY, USA) for analysis. Descriptive statistics were employed to summarize and analyse the data. Continuous variables, such as age, were presented as means and standard deviations, while categorical variables, including survey response frequencies, were reported as absolute counts and percentages of valid responses. Given the descriptive nature of the study and its aim to explore current physiotherapy practices, no inferential statistical analyses were conducted. The use of descriptive statistics was deemed appropriate for the study objectives and is consistent with the approach adopted by the original study [18] that developed the questionnaire utilized in the present research.

## 3. Results

### 3.1. Participants’ Characteristics

A total of 399 licensed physiotherapists participated in the study, with a mean age of 31.2 ± 6.6 years. The majority were male (64.2%, n = 256) and Saudi nationals (94.7%, n = 378). In terms of clinical rank, most participants were specialists (72.5%, n = 289), followed by senior specialists (18.3%, n = 73). Regarding clinical experience, 51.1% (n = 204) reported having less than six years of experience, 40.9% (n = 163) had between six and fifteen years, and 8.0% (n = 32) had more than fifteen years. Participants were employed across various healthcare sectors, with the largest proportion working in Ministry of Health facilities (42.4%, n = 169), followed by the private sector (24.1%, n = 96). A summary of participants’ characteristics is presented in Table 1.

### 3.2. Description of Physiotherapy Services

Participants reported substantial variability in access to physiotherapy services for patients with plantar fasciitis. Specifically, 28.1% (n = 112) indicated that patients typically waited less than one week, while 25.6% (n = 102) reported waiting times of one to two weeks. Longer delays were also noted, with 22.1% (n = 88) citing waiting periods of three to four weeks, and 16.9% (n = 57) indicating delays extending up to six months. In terms of referral sources, the majority of participants (82.9%, n = 331) received referrals from orthopaedic consultants. Additional referral pathways included patient self-referral (36.3%, n = 145), general practitioners (26.8%, n = 107), and rheumatology consultants (15.7%, n = 63). The duration of the initial physiotherapy assessment also varied. The most commonly reported assessment duration was 20 min (38.6%, n = 154), followed by 10 min (30.8%, n = 123). Regarding treatment session length, 30-min sessions were most frequently reported (42.9%, n = 171), followed by 40 min (23.3%, n = 93) and 20 min (19.8%, n = 79). With respect to the total number of treatment sessions provided, including the initial assessment, the most common was six sessions (38.3%, n = 153), while 23.8% (n = 95) provided more than six. Additionally, 43.4% (n = 173) of participants reported working as part of a multidisciplinary team. A comprehensive summary of these findings is presented in Table 2.

### 3.3. Diagnostic Criteria

**Figure 1:**
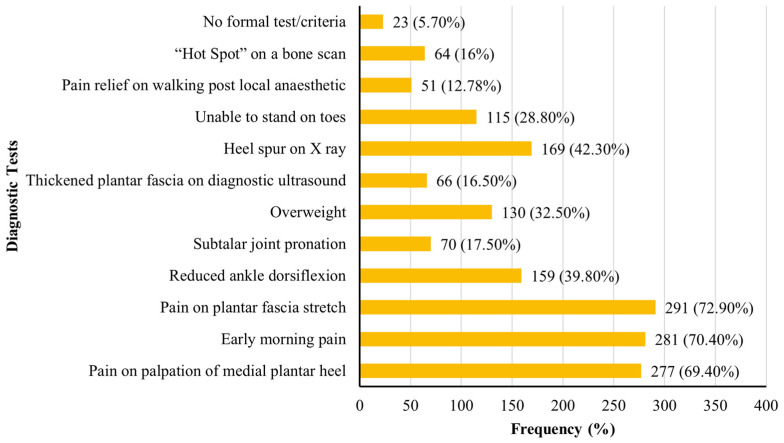
Diagnostic tests used by physiotherapists to diagnose plantar fasciitis.

Figure 1 illustrates the diagnostic criteria used by physiotherapists. The most frequently reported diagnostic tests were pain during plantar fascia stretching (72.9%, n = 291), early morning heel pain (70.4%, n = 281), and pain on palpation of the medial heel (69.4%, n = 277). Additional criteria included heel spur identification on X-ray (42.3%, n = 169), limited ankle dorsiflexion (39.8%, n = 159), and excess body weight (32.5%, n = 130). Functional limitations, such as inability to stand on the toes, were also reported (28.8%, n = 115). Less commonly used diagnostic tools included diagnostic ultrasound (16.5%, n = 66), bone scan “hot spot” detection (16.0%, n = 64), and pain relief following local anaesthetic injection (12.8%, n = 51).

### 3.4. Goals of Intervention

**Figure 2:**
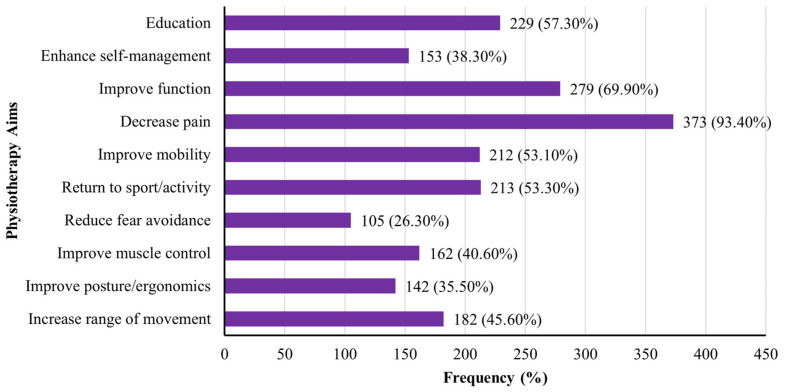
Reported aims of physiotherapy in the management of plantar fasciitis.

Figure 2 presents the goals of physiotherapy in managing plantar fasciitis. Pain reduction was the dominant aim (93.4%, n = 373), followed by functional improvement (69.9%, n = 279) and patient education (57.3%, n = 229). Other commonly reported goals included return to activity or sport (53.3%, n = 213), improved mobility (53.1%, n = 212), increased range of motion (45.6%, n = 182), and enhanced muscle control (40.6%, n = 162). Less frequently reported goals were posture and ergonomic correction (35.5%, n = 142) and reducing fear–avoidance behaviour (26.3%, n = 105).

### 3.5. Assessment of Outcome Measures

**Figure 3:**
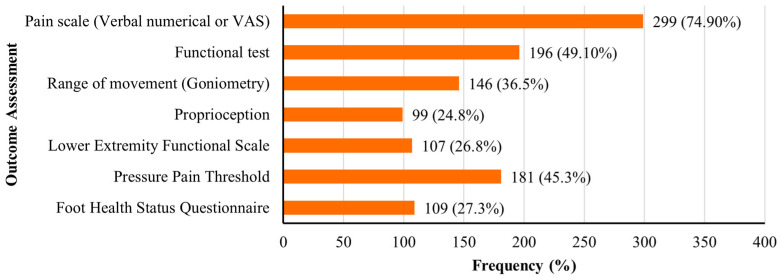
Outcome measures used by physiotherapists in the assessment of plantar fasciitis.

Outcome measures used by physiotherapists are shown in Figure 3. Pain assessment tools, such as the Visual Analogue Scale (VAS) or numerical rating scales, were the most commonly used (74.9%, n = 299). Functional performance tests were employed by 49.1% (n = 196), followed by pressure pain threshold (45.3%, n = 181) and goniometric range of motion (36.5%, n = 146). Validated patient-reported outcome measures were less frequently reported. These included the Foot Health Status Questionnaire (FHSQ; 27.3%, n = 109) and the Lower Extremity Functional Scale (LEFS; 26.8%, n = 107).

### 3.6. Intervention Strategies

Table 3 summarizes the intervention strategies applied in clinical practice. The most commonly used interventions were those reported as “always” or “frequently” used. Exercise therapy was the most frequently used approach, reported by 92.0% (n = 367) of physiotherapists. Stretching (89.4%, n = 357) and strengthening exercises (84.7%, n = 338) were also widely implemented. Patient education regarding the pathology of plantar fasciitis was reported by 75.4% (n = 301), and 81.7% (n = 326) of physiotherapists indicated that they promoted self-management strategies. Among physical modalities, shockwave therapy was used by 65.7% (n = 262), while manual therapy techniques and joint mobilizations were employed by 65.1% (n = 260) and 47.6% (n = 190), respectively. Less frequently used modalities, as indicated by responses of “rarely” or “never,” included ultrasound (48.4%, n = 193), laser therapy (24.1%, n = 96), orthotics and splints (33.1%, n = 132), hydrotherapy (50.8%, n = 203), and heat therapy (53.9%, n = 215). Dry needling and acupuncture were also infrequently applied, with 60.7% (n = 242) and 65.4% (n = 261) of participants, respectively, reporting minimal or no use.

## 4. Discussion

This national cross-sectional study is the first to comprehensively investigate physiotherapy practices for the management of plantar fasciitis in Saudi Arabia. Several key findings emerged across domains of clinical care (description of physiotherapy services, diagnostic criteria, goals of intervention, assessment of outcome measures, and intervention strategies). First, the majority of patients were referred by orthopaedic specialists, although notable variability was observed in waiting times and the duration of assessment and treatment sessions, reflecting inconsistencies in service delivery. Second, diagnosis was primarily based on clinical signs such as medial heel tenderness, early morning pain, and pain during plantar fascia stretch, while imaging techniques were infrequently used, indicating a reliance on clinical examination. Third, physiotherapists commonly identified pain reduction, functional improvement, and patient education as the primary goals of intervention, consistent with current clinical guidelines. Fourth, outcome assessment predominantly involved pain scales and functional tests, whereas standardized tools such as FHSQ and LEFS were less frequently applied. Fifth, the most commonly used interventions were therapeutic exercises, stretching, strengthening, and education, while techniques such as dry needling, acupuncture, and hydrotherapy were rarely employed. Overall, these findings indicate substantial alignment with evidence-based guidelines but also reveal areas of variability in access to care, diagnostic methods, outcome assessment, and treatment implementation that warrant further standardization and professional development.

### 4.1. Description of Physiotherapy Services

Findings revealed that patients with plantar fasciitis in Saudi Arabia are most commonly referred to physiotherapy by orthopaedic specialists. While this pathway aligns with international patterns of care [18], the study also found considerable variability in waiting times, with some patients receiving treatment within one week, while others experienced delays extending to several months. Such variation may be influenced by regional differences in service capacity, administrative processes, or healthcare infrastructure. Similarly, the duration of assessment and treatment sessions varied widely, ranging from 10 to 60 min, with a typical session lasting around 30 min. This variability in access and service structure may impact the consistency and quality of care delivered. Shorter or delayed sessions could limit comprehensive clinical evaluation, restrict implementation of tailored treatment plans, and reduce patient engagement, potentially contributing to inconsistent outcomes or prolonged recovery [25]. In comparison, the UK survey by Grieve and Palmer [18] also reported heterogeneity in session length, indicating a broader need for standardizing physiotherapy service delivery models for plantar fasciitis internationally. The limited integration of multidisciplinary care, with fewer than half of physiotherapists reporting collaborative work with other healthcare professionals, further highlights a gap in the holistic management of complex musculoskeletal conditions.

### 4.2. Diagnostic Criteria

Regarding diagnosis, physiotherapists in this study primarily relied on clinical indicators such as pain on palpation of the medial heel, early morning pain, and pain during plantar fascia stretching. These criteria are strongly supported by the APTA and American Family Physician guidelines, which recommend diagnosis based on patient history and physical examination rather than imaging [11,16]. The relatively limited use of diagnostic ultrasound, despite its high accuracy and non-invasive nature, suggests barriers such as restricted access to equipment or insufficient training [13]. In contrast, some previous studies have demonstrated greater integration of diagnostic ultrasound in clinical practice, especially in specialized or sports medicine settings [13]. The findings in this study reflect a clinical emphasis on traditional examination techniques, which are cost-effective and feasible in routine care but may lack the sensitivity needed to differentiate plantar fasciitis from other causes of heel pain. The underutilization of diagnostic ultrasound in the Saudi context may stem from broader systemic and educational challenges. These include limited availability of ultrasound machines in physiotherapy departments, the absence of structured ultrasound training in undergraduate programs, and minimal opportunities for continuing professional development in musculoskeletal imaging. Additionally, physiotherapists may lack authority to perform imaging in some healthcare settings, and national clinical protocols may not explicitly support their role in diagnostic imaging [20].

### 4.3. Goals of Intervention

The primary therapeutic aims reported by physiotherapists included pain reduction (93.4%), improvement of physical function (69.9%), and provision of patient education (57.3%). These goals are consistent with those reported in the UK [18] and reflect the priorities outlined in international guidelines [11,25]. The emphasis on education and function restoration aligns with the biopsychosocial approach to musculoskeletal care, which promotes active patient engagement, behaviour modification, and long-term self-management. Interestingly, goals such as addressing fear–avoidance behaviours and improving posture and ergonomics were less frequently cited, despite their potential relevance in chronic or recurrent plantar fasciitis. This may suggest a need for greater integration of psychosocial and ergonomic considerations into routine physiotherapy practice, as recommended by recent reviews [25].

### 4.4. Assessment of Outcome Measures

In terms of outcome assessment, pain scales (such as VAS) and functional performance tests were the most frequently used measures. These tools are widely accepted in clinical practice for their simplicity and relevance to patient-cantered outcomes [18,26]. However, the underutilization of validated instruments such as FHSQ and the LEFS indicates an opportunity to enhance the comprehensiveness and comparability of outcome data. Standardized patient-reported outcome measures (PROMs) have been recommended by international panels to monitor changes over time and facilitate inter-provider benchmarking [25]. Barriers to the use of PROMs in physiotherapy practice may include time constraints, limited training on tool selection, and unfamiliarity with interpreting patient-reported scores. Additionally, many clinical settings in Saudi Arabia may lack integrated electronic health systems that facilitate routine PROM collection and documentation. In the absence of digital infrastructure or institutional mandates, clinicians may default to simpler tools that are quicker to administer, even if less comprehensive [20,21,25].

### 4.5. Intervention Strategies

Exercise-based interventions were the most commonly employed treatment approach, including stretching, strengthening, and proprioceptive training. These findings are in agreement with current clinical guidelines [11,25] and supported by several randomized controlled trials demonstrating the efficacy of stretching the plantar fascia and posterior chain in improving pain and mobility [15,27,28]. Strengthening programs targeting both foot intrinsic and proximal hip musculature have also been shown to improve gait parameters and functional capacity in patients with plantar fasciitis [28]. The widespread use of exercise-based strategies in this study suggests a strong adherence to active, functionally oriented rehabilitation models. Education and self-management strategies were also frequently used by physiotherapists. This is encouraging, as patient education has been identified as a key component of effective musculoskeletal care, enabling individuals to manage activity levels, monitor symptoms, and understand the prognosis of plantar fasciitis [14,25]. Recommended self-management techniques, such as ball rolling and load management strategies, have demonstrated positive outcomes in previous studies and were commonly included in treatment plans in the current sample [14]. However, the study also identified underutilization of several evidence-supported modalities. For instance, extracorporeal shockwave therapy, which is recommended in clinical guidelines for chronic or treatment-resistant plantar fasciitis [11,25], was used by fewer than two-thirds of physiotherapists. Possible reasons for this include the high cost of equipment acquisition, limited availability in non-specialized or public clinics, and the absence of insurance coverage or reimbursement in many settings. Additionally, shockwave therapy often requires formal training and specific credentialing, which may not be uniformly available to all physiotherapists. The lack of national clinical protocols detailing its indications, contraindications, and dosage parameters may also contribute to uncertainty in its application [20,25]. Manual therapy and joint mobilizations were employed by only about half, despite evidence suggesting benefit in selected patient populations [17]. Possible explanations include limited access to equipment, institutional restrictions, insufficient training, or low patient demand. Similarly, low use of modalities such as dry needling, acupuncture, hydrotherapy, and heat therapy reflects both international trends and the inconsistent evidence supporting these approaches [11,25].

### 4.6. Implications

The findings of this study have important implications within the context of Saudi Arabia’s Vision 2030, which prioritizes the enhancement of allied health services and the promotion of evidence-based care [19]. The strong adherence to core physiotherapy principles, particularly in exercise and education-based interventions, reflects a positive trajectory. However, variability in the use of diagnostic tools, outcome measures, and advanced treatment modalities underscores the need for national clinical guidelines tailored to the Saudi healthcare context. Enhancing access to training in advanced interventions and promoting standardized outcome monitoring could improve care quality and consistency. Furthermore, integrating physiotherapists into multidisciplinary teams and expanding early referral pathways could address service accessibility gaps and improve patient outcomes. These findings have important implications for physiotherapy education and professional development in Saudi Arabia. Strengthening undergraduate curricula by incorporating comprehensive training in diagnostic imaging, standardized outcome measures, and guideline-recommended interventions such as shockwave therapy and manual therapy may better prepare future physiotherapists for clinical practice. In parallel, postgraduate programs and continuing professional development initiatives should reinforce these competencies among practicing clinicians to support the consistent application of evidence-based care across various healthcare settings.

### 4.7. Limitations

This study has several limitations. First, the use of a self-administered online survey may introduce response bias, including potential overreporting of evidence-based practices. The sampling strategy, based on electronic distribution, may have excluded physiotherapists from remote or underrepresented regions. Additionally, the open and anonymous nature of the survey prevented the calculation of a response rate and assessment of sample representativeness. Second, although the core content of the questionnaire was based on a previously validated English version and deemed appropriate for the target population, no additional psychometric validation was conducted in the context of Saudi Arabia. Furthermore, although the questionnaire specifically focused on plantar fasciitis, it is possible that some participants may have included patients with general heel pain rather than confirmed cases, particularly in the absence of imaging confirmation. This potential misclassification is particularly relevant given that referrals are often based on clinical presentation without imaging confirmation. Third, the study did not investigate the underlying factors influencing physiotherapists’ use of specific interventions. Factors such as equipment availability, institutional policies, clinician training, and financial or logistical constraints may play a significant role in treatment selection and implementation. Fourth, the inconsistent use of validated clinical outcome measures (e.g., PROMs) in real-world physiotherapy practice may affect the accuracy, reliability, and comparability of the reported data. Without standardized outcome tracking, variations in care quality and treatment impact may be difficult to interpret across settings. Finally, although the study provides a comprehensive national overview, it did not examine subgroup differences (e.g., by provider rank, years of experience, or healthcare sector). While the total sample size was sufficient for the main objectives, the uneven distribution across these subgroups limits the ability to draw reliable subgroup comparisons. Future studies with stratified sampling and subgroup-specific analyses are warranted.

## 5. Conclusions

This national study provides a detailed overview of physiotherapy practices for plantar fasciitis in Saudi Arabia. The results demonstrate substantial alignment with international clinical guidelines, particularly in diagnostic approaches, therapeutic goals, and the use of exercise and education-based interventions. Nevertheless, the findings also reveal variability in outcome assessment and limited use of certain evidence-based modalities. These insights highlight both the strengths and areas for improvement in current clinical practice. Future efforts should focus on promoting standardized care pathways, expanding access to training and technology, and conducting longitudinal or interventional studies to evaluate treatment outcomes. Enhancing clinical practice in the management of plantar fasciitis in Saudi Arabia requires the development of national clinical guidelines that are tailored to the local healthcare context and grounded in current evidence. At the same time, expanding access to structured continuing education programs, particularly those focused on advanced physiotherapy interventions such as shockwave therapy and manual therapy, may support greater consistency in the delivery of care. The routine use of validated patient-reported outcome measures, including the FHSQ and LEFS, should also be prioritized to facilitate more standardized and comprehensive monitoring of treatment outcomes. Strengthening the involvement of physiotherapists in multidisciplinary teams and establishing efficient referral pathways may help address existing gaps in service accessibility and coordination. At the health system level, incorporating these strategies into national rehabilitation frameworks, institutional protocols, and licensure standards could promote consistency, improve care quality, and align physiotherapy services with internationally recognized best practices. Collectively, these measures have the potential to advance musculoskeletal health outcomes and optimize the effectiveness of physiotherapy care for individuals with plantar fasciitis throughout Saudi Arabia.

## Figures and Tables

**Table 1:** Participants’ characteristics.

Characteristic		Mean ± SD or N (%)
Age		31.2 ± 6.6
Sex	Male	256 (64.2)
	Female	143 (35.8)
Nationality	Saudi	378 (94.7)
	Other	21 (5.3)
Rank	Consultant	7 (1.8)
	Senior specialist	73 (18.3)
	Specialist	289 (72.5)
	Technician	30 (7.5)
Clinical experience	<6 years	204 (51.1)
	6–15 years	163 (40.9)
	>15 years	32 (8.0)
Primary work area	Medical cities	4 (1.0)
	Military hospitals	37 (9.3)
	Ministry of health facilities	169 (42.4)
	Ministry of the interior	5 (1.3)
	Private facilities serving specific industries	12 (3.0)
	Private sectors	96 (24.1)
	Specialized hospitals	37 (9.3)
	University hospitals	26 (6.5)
	Other	13 (3.3)

SD: standard deviation; N: number; %: percentage.

**Table 2:** Characteristics of physiotherapy service delivery for patients with plantar fasciitis.

Item		N (%)
How long (on average) do individuals with plantar fasciitis wait to see a physiotherapist in your service?	<1 week	112 (28.1)
	1–2 weeks	102 (25.6)
	3–4 weeks	88 (22.1)
	1–2 months	43 (10.8)
	3–4 months	19 (4.8)
	5–6 months	5 (1.3)
	Do not know	30 (7.5)
Where do you receive plantar fasciitis referrals from?	General physician	107 (26.8)
	Orthopaedic consultant	331 (82.9)
	Patients (self-referral)	145 (36.3)
	Podiatrist/Chiropodist	24 (6)
	Rheumatology consultant	63 (15.7)
	Other	5 (1.2)
What is the duration (on average) of the first assessment?	10 min	123 (30.8)
	20 min	154 (38.6)
	30 min	71 (17.8)
	40 min	31 (7.8)
	50 min	7 (1.8)
	60 min	13 (3.3)
What is the duration (on average) of each treatment session?	10 min	16 (4.0)
	20 min	79 (19.8)
	30 min	171 (42.9)
	40 min	93 (23.3)
	50 min	23 (5.8)
	60 min	12 (3.0)
How many sessions (on average) do you offer (including the first assessment)?	1 session	8 (2.0)
	2 sessions	12 (3.0)
	3 sessions	19 (4.8)
	4 sessions	41 (10.3)
	5 sessions	37 (9.3)
	6 sessions	153 (38.3)
	>6 sessions	95 (23.8)
	Do not know	34 (8.5)
Do you work as part of a multidisciplinary team?	Yes	173 (43.4)
	No	226 (56.6)

N: number; %: percentage.

**Table 3:** Intervention strategies used by physiotherapists for plantar fasciitis.

Item	N (%)					
	Always	Frequently	Sometimes	Rarely	Never	N/A
**Education and advice**						
Education on pathology	225 (56.4)	76 (19.0)	74 (18.5)	16 (4.0)	6 (1.5)	2 (0.5)
Self-management	235 (58.9)	91 (22.8)	60 (15.0)	9 (2.3)	1 (0.3)	3 (0.8)
Advice (weight loss)	181 (45.4)	77 (19.3)	97 (24.3)	30 (7.5)	10 (2.5)	4 (1.0)
**Therapeutic exercise**						
Exercise ^1^	287 (71.9)	80 (20.1)	25 (6.3)	5 (1.3)	0	2 (0.5)
Stretching ^2^	281 (70.4)	76 (19.0)	29 (7.3)	7 (1.8)	6 (1.5)	0
Strengthening ^3^	261 (65.4)	77 (19.3)	42 (10.5)	12 (3.0)	6 (1.5)	1 (0.3)
Gait re-education	143 (35.8)	89 (22.3)	100 (25.1)	50 (12.5)	14 (3.5)	3 (0.8)
**Manual therapy**						
Soft tissue technique ^4^	151 (37.8)	109 (27.3)	83 (20.8)	34 (8.5)	19 (4.8)	3 (0.8)
Joint mobilizations ^5^	101 (25.3)	89 (22.3)	87 (21.8)	59 (14.8)	52 (13.0)	11 (2.8)
**Thermal modalities**						
Ice	160 (40.1)	79 (19.8)	108 (27.1)	28 (7.0)	22 (5.5)	2 (0.5)
Heat	32 (8.0)	44 (11.0)	94 (23.6)	67 (16.8)	148 (37.1)	14 (3.5)
**Electrotherapy modalities**						
Laser	39 (9.8)	57 (14.3)	93 (23.3)	65 (16.3)	113 (28.3)	32 (8.0)
Shockwave therapy	162 (40.6)	100 (25.1)	58 (14.5)	28 (7.0)	34 (8.5)	17 (4.3)
Ultrasound	84 (21.1)	109 (27.3)	99 (24.8)	49 (12.3)	53 (13.3)	5 (1.3)
**Hydrotherapy modalities**						
Hydrotherapy	23 (5.8)	42 (10.5)	86 (21.6)	72 (18.0)	131 (32.8)	45 (11.3)
**Assistive devices**						
Orthotics and splints	71 (17.8)	61 (15.3)	115 (28.8)	64 (16.0)	73 (18.3)	15 (3.8)
**Needling therapy**						
Dry needling	17 (4.3)	29 (7.3)	54 (13.5)	61 (15.3)	181 (45.4)	57 (14.3)
Acupuncture	9 (2.3)	14 (3.5)	41 (10.3)	47 (11.8)	214 (53.6)	74 (18.5)

N: number; N/A: not applicable. ^1^ Exercise might include functional exercise, range of movement, muscle control, proprioception, etc. ^2^ Stretching might include stretching of calf muscles, plantar fascia, hamstring muscles, etc. ^3^ Strengthening might include strengthening of calf muscles, intrinsic foot muscles, core stability, etc. ^4^ Soft tissue technique might include massage, myofascial release, myofascial trigger point therapy, specific soft tissue mobilizations, transverse frictions, etc. ^5^ Joint mobilizations might include mobilizations of ankle joint, tibiofibular joint, or subtalar joint.

## Data Availability

The original contributions presented in the study are included in the article; further inquiries can be directed at the corresponding author.

## Abbreviations

The following abbreviations are used in this manuscript:

APTA	American Physical Therapy Association
WHO	World Health Organization
STROBE	Strengthening the Reporting of Observational Studies in Epidemiology
SPSS	Statistical Package for the Social Sciences
UK	United Kingdom
FHSQ	Foot Health Status Questionnaire
LEFS	Lower Extremity Functional Scale
VAS	Visual Analogue Scale
PROMs	Patient-Reported Outcome Measures
